# Anxiety and Teacher-Student Relationships in Secondary School: A Systematic Literature Review

**DOI:** 10.1007/s10578-024-01665-7

**Published:** 2024-03-06

**Authors:** Darby Salter, Aswathi Neelakandan, Viviana M. Wuthrich

**Affiliations:** 1 School of Psychological Sciences, Macquarie University, Sydney, Australia; 2 Macquarie University Lifespan Health & Wellbeing Research Centre, Macquarie University, Sydney, Australia

**Keywords:** Anxiety, Teacher-student relationship, Adolescent, Secondary School, Teacher support

## Abstract

Anxiety disorders are the most prevalent mental disorders experienced by adolescents. As students spend a significant amount of time within a school environment, it is not surprising that factors in the school environment have been linked to student mental health. Positive teacher-student relationships (TSRs) in children have been found to improve student mental health outcomes, with supportive TSRs associated with reduced student anxiety, and in turn, student anxiety has also been associated with reduced poorer TSR quality. The findings in adolescents are less clear. This review aimed to systematically evaluate the impact of TSRs on anxiety in secondary school students, and vice-versa using PRISMA guidelines. Searches were conducted in five databases and studies screened against inclusion and exclusion criteria, and rated for study quality by two independent researchers. Twenty-six studies across 12 countries were included. Most studies reported higher quality TSRs (e.g., those that are perceived as more supportive, caring, and warm) was associated with decreased anxiety. Conversely, TSRs that were characterised by dependence, motivational support, conflict, or harassment, were associated with increased anxiety. Most studies used a cross-sectional design and as such conclusions regarding causality as well as the direction of the effects cannot be made. However, early evidence from a limited number of longitudinal studies indicated that positive TSRs reduced anxiety over time. Future research is warranted to investigate whether anxiety affects TSRs, as well as exploring specific strategies and approaches teachers can use to establish positive relationships with their students.

## Introduction

Anxiety is the most prevalent mental health disorder experienced by adolescents, with approximately 7% of young people aged between 12 to 17 years old experiencing clinically significant levels of anxiety [1]. In addition to being highly prevalent, anxiety disorders are associated with significant impairments to adolescents’ self-esteem, social competence, peer relationships, and academic performance [2], Kashani and Orvaschel [3, 4], and can increase an adolescent’s risk of developing other psychological disorders in adulthood [5]. Taken together, the high prevalence of anxiety disorders in adolescents and the associated negative consequences emphasises the need for a better understanding of the factors in adolescents’ environment which may cause and maintain anxiety during this developmental stage. To date, research on anxiety in children and adolescents has predominantly focused on the identification of risk factors in individuals (e.g., genetics, temperament, attention bias) and in the family environment (e.g., overprotective parenting and insecure attachment, [6]. Yet, students spend a significant amount of time within their school environment and thus teachers may also play an important role in the development and maintenance of anxiety beyond that of parents through positive and negative interactions with students that might increase, decrease or maintain anxiety. For example research indicates teachers use of fear appeals to motivate students to improve academic outcomes can be associated with increased anxiety Belcher et al. [7].

In addition to influencing academic outcomes, research on teacher-student relationships (TSRs) has found these relationships are a significant contributor to student mental health outcomes overall [8, 9]. In particular, TSRs that are perceived as being supportive have been associated with improved student wellbeing [10], resilience [11], self-esteem, and social skills [12, 13], and negatively associated with distress, suicidal behaviours, and depression [14], Udheim 2005, [15, 16]. Moreover, research has found that TSRs tend to become less positive as students grow older [17], which is concerning as adolescents have also been found to be more sensitive to the degree of support they receive from their teachers [18].

While the conceptual framework for TSRs has varied across the literature and has roots in education and psychology, research on the role of quality TSRs with regards to student mental health outcomes has largely been motivated by an extended attachment framework [19, 20]. According to attachment theory, early relationships between children and their parents are central to a child’s development and can provide a secure base for psychological and social development [19, 21]. Applying this theory to the school environment, teachers are believed to buffer students from developmental risks by providing them with a sense of security, and helping them relate to peers, regulate emotions, and use positive coping strategies [22]. Thus, Pianta [23] defined relationships between teachers and students as characterised by degrees of dependence, closeness, and conflict, while Murray [24] described them as characterised by warmth, trust, expectations, and involvement.

For adolescents, several conceptual models have been used to describe TSRs including attachment, developmental systems, social-motivational, and interpersonal theory [9, 25, 26]. Central to each of these theories is the importance of a sense of relatedness and involvement which includes teacher’s expression of empathy, warmth, and emotional support for the adolescent [20]. In addition, teachers are proposed to influence student motivation by increasing student’s sense of competence and autonomy [9, 25]. Thus, the term ‘positive teacher-student relationship’ is often generally used to describe when adolescents perceive that these teacher qualities are expressed towards them McHugh et al. [27]. On the other hand, negative TSRs are characterised by higher levels of conflict between teachers and students and a lack of emotional security [26].

Regarding TSRs and student anxiety specifically, although research examining this relationship in adolescents is scarce, studies based on primary-school aged children have linked positive TSRs with reduced student anxiety e.g., [28, 29], and greater teacher-student conflict with increased anxiety [30, 31]. In line with predictions from the attachment framework, Semeraro et al. [32] found that teachers who expressed more warmth, responsiveness, trust, and accessibility were better able to support students in regulating their anxiety. Further, Murray and Murray [33] found that close TSRs were associated with lower anxiety symptoms, while dependent relationships were associated with increased anxiety. Of the limited research focused on adolescents, similar findings were reported on the association between dependent teacher relationships and higher test anxiety [34]. However, findings regarding the effects of positive TSRs on anxiety are inconsistent. Keçici [35] found that positive TSRs (perceived teacher social support) were associated with reduced anxiety in female adolescents only, while Fredrick et al. [36] found a similar effect for perceived teacher support but in males. On the contrary, results from other studies found no significant effect for overall positive TSR but did for perceptions as teachers as positive motivators [34].

As these studies are predominantly cross-sectional, one of the main limitations to this research is the inability to demonstrate causation or the direction of these effects. In other words, it is equally possible that more anxious children elicit more dependent or conflictual relationships, or that students who are low in anxiety may promote more positive relationships with their teachers. In fact, theoretical models of anxiety in children and adolescents propose a bi-directional relationship between the child’s anxiety and interactions with significant others [6]. These reciprocal effects have been demonstrated in studies exploring parent–child relationships whereby anxious children are likely to elicit overprotection from their parents and, in turn, overprotective parenting is likely to lead to further anxiety [20]. As adolescence is characterised by less reliance on parents, more interactions with teachers in the secondary school environment, and an increased desire for independence and autonomy [37, 38], it is expected that greater autonomy support, rather than dependence, would lead to reduced anxiety. Teacher autonomy support is characterised by behaviours such as promoting self-directed learning and providing more opportunity for choice [39]. In a longitudinal study by Yu et al. [40], teacher autonomy support was found to reduce anxiety and depression in adolescents over time. Other studies have also reported similar findings in adolescents (e.g., [14, 41, 42], suggesting that there is considerable empirical support for the protective effect of teacher autonomy support on adolescent anxiety.

In summary, research thus far has indicated that positivity, teacher conflict, and teacher autonomy support are all important facets of TSRs that have the potential to either buffer or increase risk or severity of anxiety in students. However, as the research has predominantly focused on children in primary schools, and research in adolescents has reported inconsistent findings, the aim of this study was to conduct a systematic review of the literature to investigate the relationship between TSRs and anxiety in adolescents attending secondary school. Based on previous literature and models of anxiety, it was hypothesised that the relationship would be reciprocal, that is that factors in the TSRs would influence adolescent anxiety, and in turn, adolescent anxiety would influence factors in the TSR*.* To the best of our knowledge, this is the first systematic review on TSRs to focus exclusively on anxiety in secondary school students. It is anticipated that the results from this review will assist secondary schools in informing teachers on how to build relationships with students that can promote security and reduce anxiety.

## Method

### Search Strategy

This systematic review was conducted in accordance with the latest guidelines by the Preferred Reporting Items for Systematic Review and Meta-Analyses PRISMA; [43]. A search was conducted using the following databases,PsycINFO (1806 to 2022), Scopus (1909 to 2022), Education Resources Information Centre (ERIC; 1963 to 2021), Academic Search Premier (1972 to 2022), Education and Research (1899 to 2022), and Psychology and Behavioural Sciences Collection (1964 to 2022). For each database, all available articles were searched until the 11th of February 2022, when the final search was completed.

The search terms were selected to capture TSRs and anxiety in secondary school students, and were as follows: (((teach* or educator*) W3 *(i.e., within 3 words of)* (student* or adolesc* or teen* or pupil*)) OR (“teach* connect*” or “teach* relationship*” or “school* connect*” or “student* relationship*”)) AND (anxi* or stress* or worry or panic* or fear* or social phobi*”) AND (teen* or adolescen* or “high school” or “secondary school” or ‘secondary education”) as appeared in title, abstract and keywords. The following subject headings were also searched in the PsycINFO database: Teacher Student Interaction AND Generalized Anxiety Disorder/ or Anxiety Disorder/ or Separation Anxiety Disorder/ or Anxiety/ or Separation Anxiety/ or Social Anxiety/ or Social Phobia/ or Panic/ or Panic Disorder. Results were limited to studies written in English and published in peer-reviewed journals.

### Study Selection and Inclusion Criteria

Studies identified using the search strategy in each database were downloaded and imported into the web application Rayyan Ouzzani [44]. Prior to title and abstract screening for eligibility, duplicates were removed. Studies were required to meet the following criteria: (1) participants were secondary school students (minimum age of 11 years),(2) the focus of the study was an evaluation of the relationship between levels of anxiety and teacher-student relationships; (3) were published in the English language in a peer reviewed journal; and (4) were original research papers. In addition, for quantitative studies outcome measures included (5) students’ level of anxiety and (6) an assessment of the quality of the teacher-student relationship, and for qualitative studies (7) feedback was sought specifically on the quality of TSRs in response to student anxiety. Studies that included both primary and secondary school students were included if subgroup analyses for secondary school students were reported. Given that many countries also have adolescents attending a “middle school” with students typically starting at age 11, studies including this population were included so as to not exclude studies conducted in middle school. Studies were therefore excluded if they included university students or students below the age of 11 without reporting on subgroup analyses for the TSR and anxiety measures. Furthermore, studies that included teacher interpersonal characteristics relevant to the teacher-student relationships (e.g., teacher sensitivity and teacher support) were also included as they provided insight as to which particular qualities of the relationship may influence adolescent anxiety.

Potentially eligible articles were imported to the Covidence Systematic Review Management Software (Veritas Health Innovation, Melbourne, Australia) where they were subsequently assessed via full-text review to determine whether study criteria were met. The screening and full text review process was conducted independently by two researchers (DS and AN), with discrepancies resolved through discussion. The reference list of included articles at the full-text stage were searched for additional studies (with no new eligible studies identified).

### Data Extraction

The following data was extracted: study characteristics (authors, year of publication), sample characteristics (sample size, age, gender, type of school, country), study method (qualitative, quantitative, measures used), and outcomes (relationships between key variables and co-variables). Table 1 summarises the studies included in the review.

**Table 1:** Articles included in the systematic review

Author	Sample	Sample size (Sex)	Sample age	Measures of TSR and anxiety	Findings relevant to TSR and anxiety	Other findings for gender and culture
Aldrup et al. [49]	German secondary school students (grades 5–7)	1,559 (53% female)	Range = N/R *M* = 10.73, *SD* = 0.57	Mathematics anxiety (subscales of worry and emotionality) Teacher sensitivity scale	Students who perceived higher teacher sensitivity reported less mathematics anxiety in the subsequent year. Parents also noticed that their children’s maths anxiety decreased when they had a teacher who was aware of individual academic and emotional needs	Girls reported higher math anxiety levels No gender differences found for teacher sensitivity
Bieg et al. [66]	American and German students (Grade 8)	N = 870 (American = 425; 47% female) (German = 445; 52% female)	11–16 years *M* = 13.7 *SD* = 0.65 12–16 years *M* = 13.8 *SD* = 0.64	Perceived autonomy support Perceived teacher’s care anxiety scale	For German students, higher perceived teacher care was associated with significantly less anxiety	Compared to the German students, American students reported higher levels of anxiety, autonomy support and teacher’s care
Clem et al. [58]	Adolescents in Finland	854 (54% female)	Range = N/R *M* = 12.29 *SD* = 0.40	Student–Teacher Relationships Scale (STRS) Achievement emotions Questionnaire (AEQ; anxiety subscale)	Teacher-student conflict was negatively related to enjoyment and positively related to anxiety For students with low effortful control, closer TSRs were associated with increased anxiety towards literacy learning. For students with high effortful control, anxiety towards literacy learning did not change as a function of teacher-student closeness	
Conner et al. [61]	American high school students (grades 9–12)	5,557 (55% female)	Range = N/R *M* = 15.64 *SD* = 1.20	Academic worries scale Teacher support scale	Greater teacher support was associated with less academic anxiety, internalising symptoms and somatic symptoms related to school stress. Students who reported that more teachers supported them experienced less anxiety symptoms than those who reported that only some, a few, or none of their teachers supported them	
Federici & Skaalvik [59]	Norwegian middle school students (grades 9 and 10)	309 (52% female)	Range = N/R *M* = N/R	Scale of teacher emotional support Scale of teacher instrumental support Math anxiety Help-seeking behaviour	Instrumental support from teachers was related to reduced math anxiety and increased help-seeking behaviour Emotional support from teachers was not found to significantly relate to math anxiety	Female students showed higher help-seeking behaviours
Fredrick et al. [36]	American middle school students (grades 6–8)	169 (50% female)	Range = 11–15 *M* = 12.59 *SD* = N/R	Child and Adolescent Social Support Scale (CASS; includes teacher-support subscale) Revised Children’s Manifest Anxiety Scale, Second Edition (RCMAS-2)	Boys with high levels of teacher support reported less anxiety than boys with low levels of teacher support. A significant effect was not found in girls	Girls reported higher levels of anxiety than boys
Gairns et al. [62]	Australian High-school students (grades 7–10)	374 (100% female)	12–16 years *M* = 13.36 *SD* = 1.19	Perceived Teacher Relatedness Support (i.e., the degree to which teachers displayed interpersonally involving behaviour) Teacher-focused Relation-Inferred Self-Efficacy (RISE) appraisals Social Anxiety scale	High teacher relatedness support was linked with decreased social anxiety, via teacher-focused self-efficacy (e.g., how confident they felt their teacher was in them) and motivation	
Giota & Gustafsson [50]	Swedish secondary school students (grades 6–9)	8,603 (49% female)	13–16 years *M* = N/R	Worry (single item) Teacher relations (single item)	Positive relations with teachers in grade 6 protected against mental illness in grade 9, primarily through direct effects, but also through less stress and worry. Teacher–child conflict also contributed to internalising problems (stress and worry), which led to poor mental health outcomes	Worry increased from grade 6 to grade 9 Girls self-reported higher levels of stress and worry than boys
Guo et al. [64]	Chinese middle school students	1228 (46% female)	11–20 years *M* = 15.43 *SD* = 1.76	Depression Anxiety Stress Scale (DASS-21) Students’ Perception of Teacher’s Behavioural Support Questionnaire (SPTBSQ)	Teacher support was associated with decreased anxiety. Teacher support was also found to improve adolescent mental wellbeing by increasing resilience, affect control and help-seeking behaviours	
Hoferichter & Raufelder [56]	German secondary school students (grade 8)	513 (59% female)	13–16 years *M* = 14.03 *SD* = 0.55	Test Anxiety Inventory (TAI) Programme for International Student Assessment (PISA; teacher-student relationship subscale)	Negative correlations were observed between test anxiety and TSRs for girls only	Girls showed higher average levels of test anxiety compared to boys No gender differences were found for TSR’s
Hoferichter et al. [46]	German secondary school students Canadian secondary school students (grades 7 and 8)	1,088 (54% female) 389 (56% female)	12–15 years *M* = 13.71 *SD* = 0.53 12–16 years *M* = 13.43 *SD* = 0.82	Achievement Motivation for students (Test Anxiety subscale) PISA (teacher-student relationships subscale)	TSRs moderated the relationship between academic drive and test anxiety for German students. This moderation was not significant for Canadian students. Instead, high quality student–student relationships were found to decrease feelings of test anxiety in the Canadian sample	
Huang [69]	International high schools students in America	5,712 (50% female)	15–16 years *M* = 15.81 *SD* = 0.29	PISA (schoolwork-related anxiety and teacher unfairness subscales)	Teacher unfairness had a positive effect on schoolwork-related anxiety In addition, exposure to teacher unfairness led to increased schoolwork-related anxiety, which in turn reduced adolescent life satisfaction	
Keçici [35]	Students in Turkey (grade 8)	512 (59% female)	N/R	Neuroticism Test Anxiety Inventory (TAI) Teacher-student relationships scale (In Landauer Skala zum Sozialklima; LASSO)	Positive TSRs were associated with lower test anxiety in girls only	Girls experienced higher levels of neuroticism and test anxiety
Lapointe et al. [70]	Canadian students (grades 7 and 8)	593 (50% female)	Grade 7: *M* = 13.72; *SD* = N/R Grade 8: *M* = 14.67 *SD* = N/R	Test anxiety scale of the Motivated Strategies for Learning Questionnaire (MSLQ) Questionnaire on Teacher Interaction	For students with average grades, test anxiety reduced when maths teachers were perceived to be helping, friendly, and understanding, and increased when teachers were perceived to be uncertain and admonishing For students with high grades, test anxiety increased when maths teachers’ behaviours were perceived to be strict	Girls reported higher test anxiety than boys No gender differences found in perceptions of teachers
Lin et al. [68]	Students with Autism Spectrum Disorder in Taiwan	219 (12% female)	11–18 years Non-victims: *M* = 13.7 *SD* = 2.1 Victims: *M* = 14.1 *SD* = 2.2	Teacher Harassment Victimisation (self-report and parent-report) Taiwanese Version of the Multidimensional Anxiety Scale for Children (MASC-T)	Results indicated that victims of teacher harassment exhibited more severe anxiety than nonvictims	
Liu [57]	Chinese high school students (grade 10–12)	916 (55% female)	Range = N/R *M* = 17.6 *SD* = 0.6	Test Anxiety Inventory (TAI) School Climate scale (teacher-student relationship subscale)	TSRs negatively predicted test anxiety	Girls had higher scores on test anxiety than boys
Luo et al. [48]	Chinese high school students	950 (56% female)	13–17 years *M* = 15.1 *SD* = 1.18	Scale of Interpersonal Relationships in School (ISR; subscales measured harmonious/disharmonious relationship with teachers) State-Trait Anxiety Inventory (STAI)	Harmonious relationships with teachers were associated with decreased anxiety, particularly for students whose parents were more absent Interviews with students also revealed that those whose parents were absent had greater ambivalence in their relationships with their teachers as they were afraid of being betrayed	Influence of interpersonal relationship in schools was greater for females than males
Mainhard et al. [67]	Dutch secondary school students	1,668 (48% female)	Range = N/R *M* = 14.94 *SD* = 1.44	Academic Emotions Questionnaire (Anxiety subscale) Questionnaire for Teacher Interaction (QTI)	Teacher affection and warmth was associated with reduced student anxiety, while teacher interpersonal agency (i.e., perceived power) was related to increased student anxiety	Lower-achieving students and girls experienced slightly more anxiety than did high achievers and boys, respectively
Palmgren et al. [55]	Finnish secondary school students (grade 7)	119 (45% female)	12–14 years *M* = N/R	ECW survey (subscales measured student’s emotional engagement in teacher-student relations and school-related anxiety)	Lower quality TSRs (unfair treatment and less emotional support) were associated with greater school-related anxiety. Further, being afraid of failing (anxiety-scale) was related to perceptions of not receiving enough feedback from teacher	No gender differences found
Piechurska-Kuciel [8]	Secondary grammar school students in Poland	621 (64% females)	14–18 years *M* = 16.5 *SD* = N/R	Foreign Language Classroom Anxiety Scale School Climate-Social Action Instrumental (included a teacher support subscale)	Students who reported higher levels of teacher support experienced significantly less anxiety in their foreign language class compared to peers with lower teacher support	
Raufelder et al. [65]	German secondary school students (grade 9)	845 (55% female)	13–17 years *M* = 14.86 *SD* = 0.57	Relationship and Motivation instrument (REMO; students appraisal of their teachers as motivators) German Test Anxiety Inventory (PAF)	Test anxiety and learned helplessness was moderated by perceived teacher motivational support. In particular, high teacher motivational support was associated with increased emotionality and worry (subscales of the test anxiety measure) and decreased feelings of helplessness	
Raufelder et al. [47]	German secondary school students	88 (50% female)	Range = N/R *M* = 15.03 *SD* = 0.51	Teacher-Student Relationship Scale (TSR) Test Anxiety Questionnaire (PAF)	Students who reported a high quality TSR had stronger amygdala activity toward fearful faces, which was related to worry. Further, students with high neuroticism levels were more likely to perceive their teachers as motivators, and showed higher amygdala activity toward angry faces, which was related to emotionality	
Raufelder et al. [34]	German secondary students (grades 7 and 8)	1,088 (54% female)	12–15 years *M* = 13.7 *SD* = 0.53	Test Anxiety (subscale in the Achievement Motivation for Students) PISA (teacher-student relationship scale)	Students with higher test anxiety displayed greater socio-motivational dependence on teachers than less anxious classmates. Additionally, greater perceived positive motivation from teachers was associated with less test anxiety and more autonomous forms of academic self-regulation	
Weymouth & Buehler [51]	American middle school students	211 (51% female)	11–14 years *M* = 11.86, *SD* = 0.69	Social Anxiety Scale for Children-Revised (SASC-R) Teacher Support and Satisfaction Scale	Lower teacher support contributed to greater compliance with peers, which in turn increased social anxiety symptoms	No gender differences found
Wit et al. [63]	Canadian High school students (grades 9–10)	2,616 (54% female)	12–16 years *M* = 13.77 *SD* = 0.54	Social support for appraisals scale (SSAS; with a teacher support subscale) Generalised Social Avoidance and Distress subscale (SAD-G)	Adolescents who felt more supported by teachers experienced less social anxiety symptoms	No gender differences found
Yıldırım [60]	Students in Turkey (grades 7–12)	4,855 (43% female)	N/R	PISA (perceived teacher support scale) Maths anxiety scale	Greater perceived teacher support was associated with reduced maths anxiety	Girls reported higher levels of maths anxiety

### Synthesis Methods

A narrative synthesis only was conducted due to the significant heterogeneity in the methods used to measure anxiety and teacher-student relationships, and the variation in which teacher-student relationship characteristics were examined. In addition, as almost all studies used a cross-sectional study design, comparisons between studies in terms of effects was determined to be difficult to interpret meaningfully. Instead the focus of this study was exploration of the potential relationship between anxiety and teacher-student relationships.

### Methodological Quality

The quality of studies was evaluated using the criteria outlined in the Critical Appraisal Skills Program CASP [45] checklists for cohort studies and qualitative studies (as one study used mixed-methods). Based on the checklists, the quality of the selected studies was assessed according to five criteria,(1) examined a focused issue; (2) used an appropriate sample; (3) included outcome measures that were unlikely to be biased; (4) considered possible confounds; and (5) appropriately analysed and interpreted results. Two independent reviewers assessed risk of bias of the included articles.

## Results

**Fig. 1:**
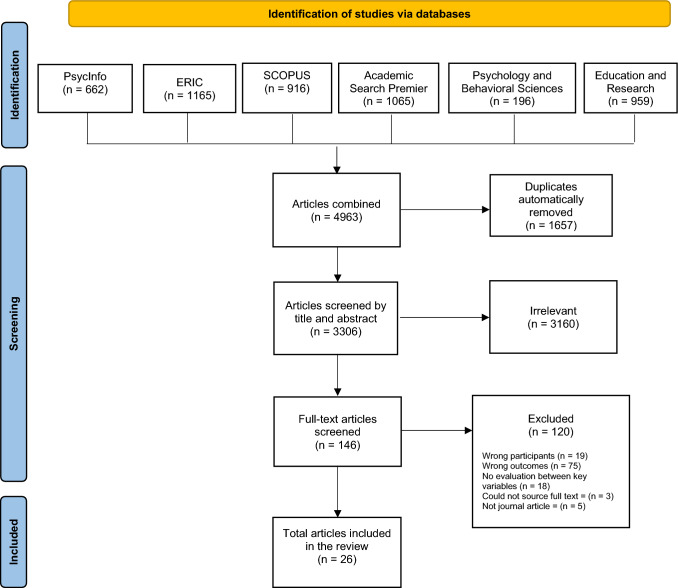
PRISMA flow diagram

See Fig. 1 for the PRISMA flowchart that depicts the results for identifying, screening, and including studies. The database search returned a total of 4963 articles, 1657 duplicates were removed, leaving 3306 articles for eligibility checking at title and abstract stage. The full text of 146 articles were reviewed, of which a total of 26 articles were eligible for the current systematic literature review.

### Study Characteristics

The 26 included studies were conducted in 12 countries including two studies which contained subsamples from two different countries. Participants included in the studies were recruited from: Germany (n = 7), United States of America (n = 5), Canada (n = 4), China (n = 3), Finland (n = 2), Turkey (n = 2), Australia (n = 1), Taiwan (n = 1), Sweden (n = 1), Poland (n = 1), Netherlands (n = 1), and Norway (n = 1). Notably, two of the studies [46, 47] used the same sample data comprising of 1088 German students, however, both studies were included in the review as their different research questions provided dissimilar outcomes. Twenty-five of the articles included in this systematic review used only quantitative methods. One used a mixed methods approach applying both qualitative and quantitative measures [48]**,** while none used only qualitative methodology. Of the quantitative studies, 22 used cross-sectional designs, three used longitudinal designs [49–51], and the mixed method study incorporated both cross-sectional analysis and interviews. Sample sizes ranged from 88 to 8603.

### Quality Assessment

The methodological quality of studies was varied, with approximately half considered to be of high quality (i.e., meeting all five criteria), and two studies [48, 50] considered to be lower quality (i.e., meeting three or less of the criteria). The most common quality limitations included using unvalidated measures or measures with only one item, over-generalising results, lacking an adequate sample (e.g., only one class or school), and failing to consider or control for potential biases (e.g., principal’s selecting which teachers and classes to include in the sample). Table 2 presents the quality ratings for each study.

**Table 2:** Quality ratings of articles in the systematic review

Author	Focused question	Adequate sample	Unlikely measurement bias	Appropriate design/confounds considered	Adequate analysis/interpretation
Aldrup et al. [49]	Yes	No	Yes	Yes	Yes
Bieg et al. [66]	Yes	Yes	No	Yes	Yes
Clem et al. [58]	Yes	Yes	Yes	Yes	Yes
Conner et al. [61]	Yes	Yes	Yes	Yes	Yes
Federici & Skaalvik [59]	Yes	Yes	Unclear	Yes	Yes
Fredrick et al. [36]	Yes	Yes	Yes	Yes	Yes
Gairns et al. [62]	Yes	Yes	Unclear	Yes	Yes
Giota & Gustafsson [50]	Yes	Yes	No	Yes	No
Guo et al. [64]	Yes	Yes	Yes	Yes	Yes
Hoferichter & Raufelder [56]	Yes	Yes	Yes	Yes	Yes
Hoferichter et al. [46]	Yes	Yes	Yes	Yes	Yes
Huang [69]	Yes	No	Yes	Yes	Yes
Keçici [35]	Yes	Yes	Yes	Yes	Yes
Piechurska-Kuciel [8]	Yes	Yes	Yes	Yes	Yes
Lapointe et al. [70]	Yes	Yes	Unclear	Yes	Yes
Lin et al. [68]	Yes	Yes	No	Yes	Yes
Liu [57]	Yes	Yes	Yes	Yes	Yes
Luo et al. [48]	Yes	Unclear	No	Yes	No
Mainhard et al. [67]	Yes	Yes	Yes	Yes	Yes
Palmgren et al. [55]	Yes	Yes	Yes	Yes	Yes
Raufelder et al. [65]	Yes	Yes	Yes	Yes	Yes
Raufelder et al. [47]	Yes	Yes	Yes	Yes	Yes
Raufelder et al. [34]	Yes	Yes	Yes	Yes	Yes
Weymouth & Buehler [51]	Yes	Unclear	Yes	Yes	Yes
Wit et al. [63]	Yes	Yes	Yes	Yes	Yes
Yıldırım [60]	Yes	Yes	Yes	Yes	Unclear
*Qualitative study*	Focused question	Adequate sample	Unlikely bias	Appropriate design/ confounds considered	Adequate analysis/interpretation
Luo et al. [48]	Yes	Unclear	Unclear	Unclear	No

Yes = met criterion, No = did not meet criterion, Unclear = unclear if criterion were met

### Outcome Measures

All studies used self-report measures as the central means of data collection. Across the studies, a variety of self-report instruments were used to assess anxiety and the quality of TSRs. Approximately 24 different self-report anxiety scales were used, of which the most commonly used measure was the Test Anxiety Inventory TAI, [52] used in three of the studies. In addition to test anxiety, other forms of anxiety assessed included maths anxiety, worry, academic/school-work related anxiety, trait anxiety, and social anxiety. Regarding TSR measures, 21 different scales were used, of which the most common were the teacher-student relationships subscale in the Programme for International Student Assessment (PISA, [53] and the Questionnaire for Teacher Interaction QTI, [54]. In addition to assessing TSRs broadly, studies often included assessments of other constructs related to teacher support, care, harassment, unfairness, and sensitivity.

### Main Findings from Studies

#### Teacher-Student Relationships

Of the 10 studies that measured the overall global quality of the relationship between students and teachers, seven suggested that higher quality global TSRs were associated with reduced student anxiety. This relationship was most consistent in studies measuring school-related anxiety [55], and test anxiety [35, 56, 57]. Results from the longitudinal study by Giota and Gustafsson [50] indicated that positive relations with teachers in grade 6 protected against mental illness in grade 9, primarily through reduced levels of stress and worry. Additionally, it was found that teacher–student conflict contributed to stress and worry, which resulted in poorer mental health outcomes. However, as this study was of poorer quality it is unclear how robust these findings are. Conversely, results from the other three studies were mixed, with one study finding no significant direct effect between TSRs and test anxiety [34], and two studies finding that a positive TSR was linked with increased anxiety levels [47, 58].

#### Teacher Support

Ten studies measured teacher support specifically. Nine of these found greater teacher support was associated with lower anxiety symptoms. Across these studies, teacher support was found to reduce foreign language anxiety [8], maths anxiety [59, 60], test anxiety [61], academic anxiety [61], and social anxiety [51, 62, 63], while also increasing resilience, affect control, and help-seeking behaviours [64]. Further, Conner et al. [61] found that students who felt more supported by their teachers also experienced fewer internalising and somatic symptoms related to school stress. Whilst these studies were all limited to cross-sectional findings and thus unable to indicate direction, research by Weymouth and Buehler [51] found in a sample of American students, lower teacher support led to greater compliance with peers 1 year later, which subsequently increased symptoms of social anxiety the following year.

#### Other Teacher Characteristics

The remaining seven studies examined the relationship between a range of other specific teacher characteristic and student anxiety. Compared to ‘teacher support’ and ‘teacher autonomy support’ which as reported above were associated with lower student anxiety, two studies found that greater socio-motivational dependence on teachers was associated with increased test anxiety and feelings of helplessness [34, 65]. This suggests that this type of teacher support which is related to student motivation may have a unique and opposite impact on student anxiety in which anxiety is increased, not decreased. The other studies examined a range of other teacher characteristics and found lower anxiety levels in students who perceived high teacher sensitivity [49], care [66], affection and warmth [67], and when they perceived their teachers to be helping, friendly, and understanding [51]. In a very large (n = 8603), high quality, longitudinal study by Aldrup et al. [49] students who perceived higher teacher sensitivity reported less maths anxiety in the subsequent year, as reported by both the students and their parents. Further, higher anxiety levels were reported in students who perceived greater teacher harassment [68], teacher conflict [58], teacher interpersonal agency (i.e., perceived power, [67], and when they were viewed as unfair, strict, uncertain, and admonishing [69, 70].

#### Gender Differences

Across nine of the studies, mean anxiety levels were found to be significantly higher in girls than boys. Although no differences were found between genders when describing average scores on the TSR measures, some significant differences appeared when examining the interactions between the anxiety and TSR variables. Two studies found that more positive TSRs were associated with lower test anxiety in girls only [35, 56] while another study found that teacher support buffered the relation between maladaptive perfectionism and anxiety in only boys [36]. In other words, for boys that were more likely to be driven by a fear of failure or disappointing others, higher teacher support was able to help lower their levels of anxiety.

#### Cultural Differences

As the studies varied widely in their focus on constructs and measures for assessment, comparisons between studies regarding cultural differences was difficult. However, studies that included samples from more than one country provided some insight into how teacher-student relationships and anxiety may vary cross-culturally. Hoferichter et al. [46] included samples of both German and Canadian students and found for the German students, higher quality TSRs were associated with increased test anxiety, whereas for the Canadian sample, no link between TSR and adolescent anxiety was found. In another study which included both German and American samples, students from the United States reported experiencing higher amounts of anxiety, autonomy support, and teacher care than the German students [66]. Further, higher teacher care was related to lower anxiety in the German students only.

## Discussion

This systematic literature review aimed to examine the relationship between TSRs and anxiety in adolescents attending secondary school. Based on previous literature, theories of TSRs, and models of anxiety, it was hypothesised that the relationship would be reciprocal, that is that factors in the TSR would influence adolescent anxiety in secondary school, and in turn, adolescent anxiety would influence factors in the TSR. Most studies either assessed the quality of the TSR using broad measures that assessed a range of relationship domains and characteristics or focused on one specific characteristic, and whilst there was great variability in the characterises of the TSR measured in general, the research suggested that TSRs that were perceived as positive and supportive were associated with reduced anxiety in adolescents attending secondary school. This is consistent with prior research based on primary-school aged children [28, 29]. Across the studies, in general lower anxiety was associated with a broad range of positive and supportive TSR characteristics such as sensitivity, care, affection, and warmth, and when students perceived their teachers to be helping, friendly and understanding. Conversely, higher anxiety levels were reported in students who perceived higher teacher harassment and conflict, and when teachers were perceived as unfair and strict. These findings are consistent with attachment theory and prior research [71]. Emotionally warm and supportive relationships between teachers and students are thought to provide the student with a sense of relatedness and security within the school environment, thus reducing symptoms of anxiety. In contrast, the psychological need for relatedness is unlikely to be fulfilled in TSRs that are characterised by a high level of conflict, increasing their risk for anxiety. Similarly, this finding also fits with research on characteristics of the parent–child relationship that have been found to be associated with reduced child anxiety, namely, warmth and low aversiveness [72].

It is important to note that 23 of the 26 included studies used a cross-sectional design, and therefore while there is evidence that the nature of TSR is associated with student anxiety, interpretations about causation cannot be made. Findings from three longitudinal studies provide some insights as to possible causal explanations. All three studies found some evidence that the nature of the TSR was associated with subsequent anxiety levels in adolescents attending secondary school, but due to differences in methodology it is unclear what aspect of the TSR might be particularly important, and also whether the relationship was bidirectional. Whilst the study by Giota and Gustafsson [50] was of lower quality, the studies by Weymouth and Buehler [51] and Aldrup et al. [49] were higher quality, with the later being conducted in a very large sample, suggesting that the overall finding of higher quality TSR leading to lower student anxiety over time is likely a robust finding. More research is needed to understand the causal role of TSRs, and if there are specific TSR characteristics that most strongly influence anxiety symptoms in students over time, as this could have impacts on how teachers manage relationships with students over time and reduce future student anxiety.

An interesting finding from this review was that teacher support might have a divergent association with student anxiety, depending on whether the support promotes greater student dependence or autonomy. Compared to ‘teacher support’ and ‘teacher autonomy support’ which were reported to be associated with lower anxiety in all studies that examined these factors, the need for greater ‘teacher motivational support’ was associated with increased test anxiety and feelings of helplessness [47, 65]. These findings may suggest that when teachers are perceived as motivators, this can induce feelings of expectations and pressure to perform (i.e., increased test anxiety) while also increasing dependency on the TSR for support due to decreased self-efficacy. Hence, support from teachers may not only be characterised by a personal relationship but also by expectations of educational results. Some recent research in senior students aligns with this finding. In a large sample, student anxiety was greater in students who reported lower self-efficacy and more frequent use of fear appeals by their teachers in which their teachers would emphasise the negative consequences of poor examination performance in an attempt to motivate students to study [7]. Thus, consistent with theoretical models of anxiety which propose that overprotective parenting leads to further anxiety [20], TSRs that are characterised with greater involvement and dependence may increase adolescent anxiety in secondary school settings, whereas teacher autonomy support appears to have a protective effect. However, as noted above, these findings are based on cross-sectional studies and therefore conclusions regarding causal directions cannot be made. Future research should examine the impact of teacher motivational support on student anxiety over time for further understand this association.

Studies included in this review also revealed some effects of gender on anxiety levels. For example, nine of the studies reported that anxiety levels were found to be higher in girls than boys replicating other studies [73]. These results are likely reflective of prevalence rates given that girls are often recognised as being more likely to develop symptoms of anxiety than boys [6]. Furthermore, although it has been proposed in some studies that girls report closer relationships with their teachers than boys and are more reliant on them for social support [74, 75], overall results from the current review did not indicate sex differences in terms of TSRs suggesting that boys and girls perceive similar levels of support from teachers. Similarly, with the exception of two papers [35, 56], there were no studies within the review that found gender differences when examining the relationship between anxiety and TSRs. While it is unknown why two of the studies found a significant association between TSRs and anxiety in girls only, possible explanations may relate to the higher proportion of females than male participants within both studies.

Another interesting finding stems from the two papers that included samples from more than one country. In both studies, the perceived quality of TSRs was linked with anxiety levels in the German samples only, with non-significant results found in the American and Canadian samples, respectively [46, 66]. Notably, results from one study suggested that anxiety levels were lower in German students with higher teacher care [66], while the other suggested that test anxiety levels were higher in German students who reported higher quality TSRs [46]. One possible consideration for these different results is that the study by Hoferichter et al. [46] measured test anxiety rather than trait or state anxiety. As previously described, teacher motivational support has been linked with increased test anxiety (e.g., [65]. Thus, these findings could reflect that for some students, increased support from their teacher may induce anxiety more specific to doing well in that subject (i.e., increased test anxiety). Further, the differing results across the German, Canadian and American samples was surprising given they are western cultures that are interested in promoting autonomy. However, there are also notable differences in the schooling system between these countries. For example, unlike in America or Canada, German students are often taught by the same teachers for multiple years, providing a greater opportunity for stronger relationships to develop. As the association between TSRs and anxiety within other American and Canadian samples included in the study was reported to be significant e.g., [51, 61, 63], further research investigating how teacher relationships may differ across cultures is needed to better understand this relationship across different school systems.

This review has several limitations. First, in order to balance the differences in secondary school models between countries and to be inclusive of middle school students, some children aged 11 years of age (pre-adolescent) were also included. Secondly, a meta-analysis was not conducted and so results are based on a narrative synthesis of the literature only. Finally, the systematic search was limited to articles published in English limiting generalisability to non-English speaking countries or regions. These limitations reduce the generalisation of the results. Perhaps more significantly, it is important to consider the challenges of consolidating research in this field due to the wide range of definitions and measurement of TSRs, as reviewed by Phillippo et al. [76], and the overreliance of student-reported perceptions of teacher behaviours. Further the variation in definitions and measures limits our understanding of what particular factors in the TSR are key in influencing student anxiety, and which types of anxiety are influenced by these. Thus, it is important to develop alternative approaches for assessing TSRs as having multiple informants and methods is consistent with best practice in assessment, and could help to reduce the inherent biases related to relying on one informant Murray and Zvoch [77]. Despite these challenges, the evidence across these measures was generally consistent in finding that features of TSR were associated with increased and decreased levels of anxiety in students. Although most of the studies in this review used cross-sectional designs limiting interpretations regarding causality, the three longitudinal studies found similar results and suggest that TSRs may be able to influence student anxiety levels over time, despite differences in study methods and measures. With consideration to the above limitations and challenges in the field, future research may benefit from addressing these by: (1) collecting information from multiple informants (e.g., including additional teacher ratings) to minimise bias and also capture perceptions of both student factors which can influence TSR quality, (2) utilising validated TSR measures which contain items relating to both teacher and student characteristics, (3) conducting longitudinal studies to evaluate the effect of adolescent anxiety and student behaviours (e.g., help-seeking) on TSRs; and (4) controlling for covariates such as gender and cultural background given the reported differences in anxiety levels.

## Summary

The current systematic review explored the literature on teacher-student relationships and anxiety in adolescents attending secondary school. In general, the findings indicated that TSR relationships that were higher in support, lower in motivational support and lower in conflict and dependence were associated with less student anxiety. Existing research on teacher support has thus far suggested that teachers could improve students’ sense of autonomy by providing more opportunities for choice, promoting independent decision making, minimising use of shame-inducing teaching strategies, and by acknowledging the students’ feelings e.g., [40, 78, 79]. Further research exploring the effects of these strategies on adolescent anxiety in secondary school settings could yield important information to refine recommendations for schools and assist teachers in establishing high quality relationships with their students. As most studies used a cross-sectional design, conclusions regarding causation and whether the relationships between TSR and student anxiety is reciprocal could not be made. However, consistent findings from longitudinal studies indicated that more positive TSR was associated with reduced student anxiety over time, offering promising targets for future interventions. This review therefore highlights a clear need for future studies to incorporate multiple methods and informants to collect data, as well as a greater focus on how adolescent anxiety may impact the TSR bidirectionally. Furthermore, more research on the specific teacher behaviours and approaches that are beneficial in promoting student mental health will better inform recommendations for teachers in secondary schools on how to develop quality relationships with their students.

## Data Availability

The data is freely available in the published papers included in this review. The collated data is available by request from the corresponding author.
